# A Case Report of Rattlesnake Musk Exposure Causing Chemical Conjunctivitis

**DOI:** 10.5811/cpcem.38049

**Published:** 2025-05-01

**Authors:** Raj Patel, Melanie M. Randall

**Affiliations:** Riverside University Health System, Department of Emergency Medicine, Moreno Valley, California

**Keywords:** rattlesnake, snake musk, ocular exposure, chemical conjunctivitis, case report

## Abstract

**Introduction:**

Rattlesnakes are pit vipers belonging to the *Viperidae* family and *Crotalinae* subfamily. They inject venom into their victims via bites from two long, hollow fangs. When disturbed, they can release a liquid musk from cloacal scent glands into the air. This report describes a rare case of non-penetrating rattlesnake ocular exposure with symptoms. We also discuss pathophysiology, evaluation, and treatment recommendations.

**Case Report:**

A 56-year-old male picked up a rattlesnake and was sprayed in both eyes with liquid emanating from the snake. He was not bitten by the snake. He had immediate pain and blurred vision. Despite copious initial irrigation, he continued to have worsening symptoms with subconjunctival hemorrhage and scleral injection. After discussion with poison control, he was given six vials of intravenous (IV) antivenom. After additional irrigation and evaluation by ophthalmology, the patient’s symptoms stabilized, but his exam still included blepharitis, subconjunctival hemorrhages, and bilateral, small corneal epithelial defects. He was discharged home with corneal antibiotics and artificial tears. One week later his symptoms were resolved, and his exam was normal.

**Conclusion:**

Non-penetrating musk ocular rattlesnake exposure is rare. In certain conditions, it may be from exposure to snake musk from scent glands and not venom. It should be treated as any other ocular exposure beginning with copious irrigation and then a detailed examination. Current recommendations argue against IV antivenom administration.

## INTRODUCTION

Venomous snakes use their venom for a variety of purposes including hunting, digestion, and defense.^1^ The venom can be released either by biting or spitting. Rattlesnakes are pit vipers belonging to the *Viperidae* family and *Crotalinae* subfamily. This subfamily also includes cottonmouths and copperheads.^2^ They evolved to inject venom into their victims via bites from two long, hollow fangs. According to a recent study by Maciulewicz et al, rattlesnake envenomation requires a specific angle and compression of fangs, leading to their conclusion that the likelihood of a rattlesnake spitting venom is close to zero.^3^ In contrast, there are numerous reports of a separate family of snakes, the *Elapidae* family, causing non-penetrating ocular envenomation.^4^ These snakes are mostly found outside the United States and include mambas, cobras, taipan, kraits, and coral snakes. The *Elapidae* use specialized glands that do not require compression to spit venom onto their prey. 5

Snakes that are disturbed can forcefully expel an airborne “musk” from their cloacal area via scent glands.^6^ This has been observed by herpetologists in many types of snakes including rattlesnakes.^7^ Snake musk includes multiple volatile and nonvolatile substances that are not well described.^8^ Maciulewicz et al proposes this as the likely mechanism for ocular irritation when a rattlesnake has been handled and releases liquid but no biting-down force of the mouth or fangs has occurred.^3^ Snake musk exposure is an extremely rare topic in the medical literature. This topic is primarily described in animal and zoological texts.

In this report we describe a rare, rattlesnake ocular exposure, likely from snake musk, resulting in a chemical conjunctivitis. We highlight pathophysiology, evaluation, and treatment recommendations.

## CASE REPORT

A 56-year-old male with a history of diabetes mellitus and hypertension picked up a rattlesnake with the intent to decapitate it. Instead, he was sprayed in both eyes with liquid from the snake, resulting in immediate pain and blurry vision. The patient was able to confirm that it was a rattlesnake, but no further identifying characteristics were available. At the initial hospital, poison control recommended irrigation, analgesia, ophthalmology evaluation, and topical antibiotics. Labs included complete blood count, complete metabolic panel, and coagulation studies, which were normal. Despite two liters of normal saline irrigation to each eye, his clinical examination worsened with subconjunctival hemorrhages and scleral sloughing. After a secondary discussion with poison control, he was given six vials of the intravenous (IV) antivenom CroFab (BTG International Inc, West Conshohocken, PA). As the initial hospital did not have emergency ophthalmology consultation, he was transferred to our facility.

At our emergency department he reported bilateral ophthalmalgia, subconjunctival injection, watery discharge, and blurry vision. Initial examination was significant for a visual acuity of 20/50 in the left eye, 20/70 in the right eye, mild scleral inflammation, bilateral subconjunctival hemorrhages, and bilateral watery/tan discharge (Image).

Each eye was irrigated with an additional two liters of normal saline prior to formal ophthalmology evaluation. Examination by the ophthalmologist was significant for visual acuity of 20/25 in both eyes, bilateral ocular pH of seven (normal range 7.0–7.4), blepharitis, subconjunctival hemorrhages, and bilateral small corneal epithelial defects. The anterior and posterior segments were normal except for the conjunctival hemorrhages and scleral injection.

**CPC-EM Capsule**

What do we already know about this clinical entity?

*Recent literature shows that rattlesnakes are unable to spray venom unless under very particular circumstances. Rattlesnakes can, however, release musk from cloacal glands into the air*.

What makes this presentation of disease reportable?

*Snake musk exposure is extremely rare in medical literature. We present this case of non-penetrating rattlesnake exposure causing chemical conjunctivitis*.

What is the major learning point?

*Non-penetrating rattlesnake exposure with conjunctival symptoms may be from cloacal snake musk. It should be treated with copious irrigation and examination*.

How might this improve emergency medicine practice?

*If any providers have similar patients in the future, they will be able to confidently treat as a chemical exposure and not as a rattlesnake envenomation*.

He was discharged with seven days of the following medications: ciprofloxacin eye drops four times per day, erythromycin ointment nightly, and preservative-free artificial tears every hour. The medications were chosen by the ophthalmology consultant. One week later, the follow-up examination in the ophthalmology clinic showed complete resolution of his corneal abnormalities.

## DISCUSSION

This is a rare case of non-penetrating, ocular, rattlesnake exposure, presenting with corneal defects and subconjunctival hemorrhage. Previously, Troutman et al and Cantrell et al both described cases with mild symptoms treated to resolution with irrigation only.^9^,^10^ Johnson described a case treated with irrigation, IV antivenom, and topical antibiotics.^11^ In these case reports it was assumed the symptoms were from rattlesnake envenomation. In our case, the treating clinicians initially considered this to represent another rare case of rattlesnake non-penetrating envenomation. However, given the recent study by Maciulewicz et al demonstrating rattlesnake spitting venom as extremely unlikely in these conditions, we feel this more likely represented a chemical conjunctivitis from exposure to snake musk

Most documented snake ocular exposures are from African and Asian *Elapidae* spitting snakes. All published data demonstrates anterior ocular segment damage including subconjunctival injection, blepharitis, corneal erosions, and uveitis.^4^,^5^ Delayed time to treatment has been shown to result in complications such as corneal opacities and blindness. *Elapidae* venom has effects similar to chemical burns in the eye. Inflammatory cytotoxic compounds such as metalloproteinase and phospholipase are the culprit venom components. There are no reports of systemic toxicity from ocular snake-venom exposure.^4^,^5^

The treatment of any ocular substance exposure is immediate and prolonged irrigation. Additional options include topical analgesics, topical antibiotics, cycloplegics, and antihistamines.^4^,^5^ Evaluation should include pH testing, visual acuity evaluation, slit lamp examination, and fluorescein staining. Emergency ophthalmology evaluation may be necessary if symptoms worsen or do not resolve. Topical corticosteroids, topical antivenom, and IV antivenom are contraindicated due to potential adverse effects and lack of evidence showing systemic toxicity.^4^,^5^,^12^

In our case, IV antivenom was empirically recommended by our local poison center when the patient’s symptoms did not initially improve. After reviewing the most recent literature, it is likely that continued supportive care of the patient’s chemical conjunctivitis, rather than antivenom treatment, resulted in symptom resolution.

## CONCLUSION

Non-penetrating, ocular rattlesnake envenomation is a rare occurrence. Under the right conditions, exposure may be due to musk released from snake scent glands, causing a chemical conjunctivitis. It should be evaluated and treated as any other ocular exposure beginning with copious irrigation and then a detailed examination. Current recommendations advise against intravenous antivenom administration.

## Figures and Tables

**Image:**
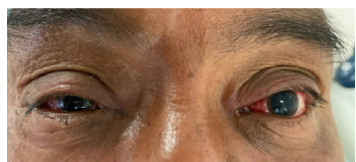
Patient with mild scleral injection and bilateral subconjunctival hemorrhages after rattlesnake musk exposure.
